# Promise and performance of agricultural nutrient management policy: Lessons from the Baltic Sea

**DOI:** 10.1007/s13280-021-01549-3

**Published:** 2021-05-27

**Authors:** Martin Hvarregaard Thorsøe, Mikael Skou Andersen, Mark V. Brady, Morten Graversgaard, Emils Kilis, Anders Branth Pedersen, Samuli Pitzén, Helena Valve

**Affiliations:** 1 Department of Agroecology, Aarhus University, Blichers Alle 20, Foulum, 8830 Tjele, Denmark; 2 Department of Environmental Science, Aarhus University, Nordre Ringgade 1, 8000 Århus C, Denmark; 3 AgriFood Economics Centre, Department of Economics, Swedish University of Agricultural Sciences (SLU) and Centre for Environmental and Climate Science (CEC), Lund University, Box 730, 220 07 Lund, Sweden; 4 Baltic Studies Centre, Kokneses prospekts 26-2, Riga, 1014 Latvia; 5 Finnish Environment Institute (SYKE), Latokartanonkaari 11, 00790 Helsinki, Finland; 6 Department of Environmental Science, Aarhus University, Frederiksborgvej 358, Risø, 4000 Roskilde, Denmark

**Keywords:** Agri-environment, CAP, HELCOM, Marine policy, Policy instrument, Rural development

## Abstract

Following decades of international collaboration to restore the Baltic Sea, we provide an assessment of the domestic implementation of measures agreed to limit diffuse agricultural pollution and the patterns of policy instruments applied. Despite the Helsinki Convention being unusually specific in detailing what measures countries should introduce, we find many shortcomings. These are most pronounced in the larger countries (Poland, Germany and Russia), while smaller countries perform better, notably Sweden and Estonia. The patterns of policy instruments applied differ, influenced by domestic politics. The limited use of complementary policy instruments suggests that other priorities overrule full and effective implementation, with engagement mirroring the advantages that a restored Baltic Sea can bring to countries. Using the European Agricultural Fund for Rural Development to support farmers in managing nutrients, particularly advisory services and investments in modern manure management technologies, represents a significant opportunity for reducing agricultural pollution in most countries.

**Supplementary Information:**

The online version contains supplementary material available at 10.1007/s13280-021-01549-3.

## Introduction

**Fig. 1:**
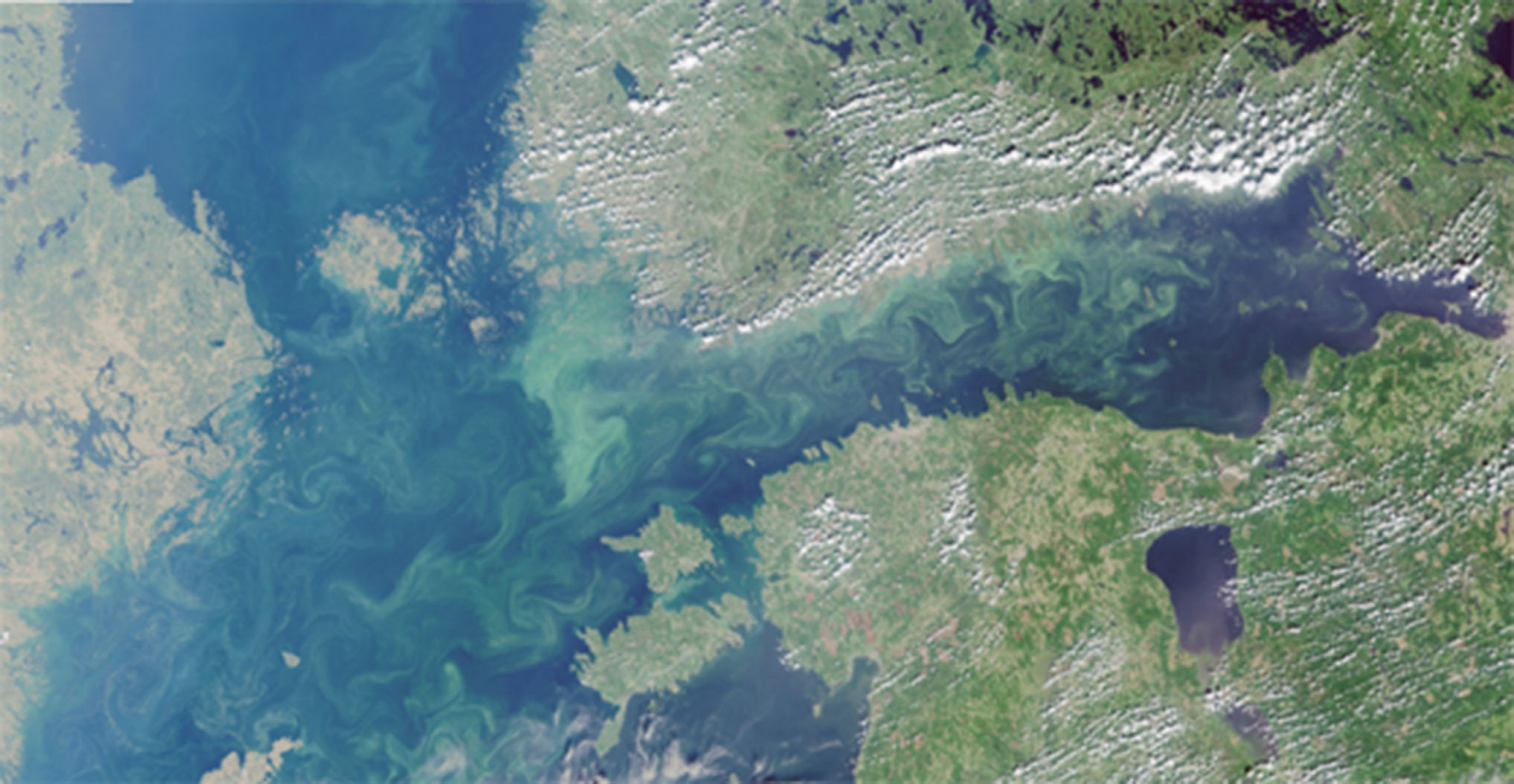
Algal blooms. Eutrophication situation on 16 July 2018 in the Finnish coastal waters of the Baltic Sea. *Source* ESA Copernicus Sentinel Data

The Baltic Sea is the largest body of brackish water in the world, and as the shallow Danish straits tend to limit its outflow and water exchange, its residence time of 35–40 years results in the accumulation of nutrients discharged from a large region. Despite more than four decades of international collaboration, 97% of the Baltic Sea continues to suffer from eutrophication, involving phytoplankton growth, reduced light conditions, oxygen depletion and a high frequency of toxic algal blooms (HELCOM 2018b) (Fig. 1). Diffuse pollution via rivers and the atmosphere, originating mainly from agriculture, accounts for 60–65% of the anthropogenic loads of nitrogen (N) and phosphorus (P) from the littoral countries (EMEP 2013; HELCOM 2018a). Over the past 25 years pollution has declined by 14% for N and 24% for P, mainly due to reductions of point source pollution. The annual loads are still exceeding the Maximum Allowable Inputs defined by the Baltic Sea Action Plan (BSAP) by about 100 000 tonnes N and 8000 tonnes P, corresponding to 13% and 38% for N and P, respectively (ibid.). Moreover, model predictions suggest that by 2050 and solely due to climatic changes, 8–14% increases in the nutrient loads should be expected (Øygarden et al. 2014; Bartosova et al. 2019).

The Helsinki Convention on the Protection of the Marine Environment of the Baltic Sea Area^1^ (henceforth the *Convention*) was agreed in 1974 as a pioneering framework for east–west collaboration on restoration. Stringent measures for agricultural nutrient management were defined and agreed by the Convention Parties in 1998 and amended in 2007 (Kremser 1997). What is special and remarkable about this part of the Convention, is that the littoral countries committed themselves to implement the specified provisions into their national regulations.^2^ Following the collapse of the planned economies, all but one of the post-Communist countries (Russia) have joined the European Union (EU), and become subject to the EU *acquis*. The Convention and its Commission (HELCOM) nevertheless is regarded as an important vehicle for concerted action, including for cooperation with Russia, providing in fact “a legally binding agreement” (Bohman 2017, p. 122) with a stronger judicial status than the BSAP and its country-allocated reduction targets. Although the measures addressing agriculture are listed in an annex, such annexes form according to Article 28 an integral part of the Convention. Moreover, where there are “specified requirements levels” they are according to the annex stated to be “a minimum basis for national legislation”.

The second holistic assessment published by HELCOM (2018b) makes note of poor implementation of measures addressing eutrophication, but provides no details about the specific shortcomings. HELCOM relies on Parties to report their domestic efforts, without recording the measures actually implemented or their relative effectiveness (Bohman 2018). While there are studies of nutrient management in individual countries (Dalgaard et al. 2014; Drangert et al. 2017; Kowalczewska et al. 2018), a systematic comparative analysis and assessment of whether the agreed measures to control agricultural pollution have been implemented has so far not been undertaken.

The objective of this article is therefore to map the national level compliance with the agreed Convention measures and the associated policy instruments employed to limit agricultural nutrient pollution, with the aim of analyzing and characterizing patterns of domestic implementation within the context of a common framework of obligations. We believe this analysis to be timely in view of HELCOM’s call for improving implementation and the 2021 review of the BSAP.

## Theoretical framework

As an international environmental agreement, the Helsinki Convention provides an opportunity to compare compliance within a common framework of obligations and to study domestic implementation across different countries. Domestic implementation refers to the long-term process of converting international commitments, reflecting formal agreement of governments, into national policies and measures as well as ensuring behavioral changes of target groups (Skjærseth 2000).

While changing the behavior of target groups may require many years, obtaining improvements in water quality will take even longer. Thus it is essential to differentiate between the *output* and the *outcome* of agreements; while *outcome* refers to the substantive changes obtained, e.g. in emissions reductions and environmental quality, *output* refers to the formal aspects of translating an agreement into decisions at the domestic level. Legal scholars conventionally refer to the latter process as the transposition of supranational decisions into national law (Bohman 2017). While the *outcome* can be influenced by unexpected economic and biophysical factors, the domestic implementation of *output* can be expected to reflect more closely the willingness and ability of national level decision-makers to honor international commitments.

The conventional view that ‘almost all countries comply with almost all their international commitments’ (Henkin 1968, p. 45) stems from the assertion that countries will be very conservative in what binding international commitments they adopt, in part due to the inability to secure certain outcomes. However, with the advent of globalization and Europeanization the number of international agreements and commitments have multiplied and some countries appear nowadays to be less cautious in what they sign up to, especially where financial resources can be obtained or security interests are at stake. Four different modes of domestic implementation of international environmental agreements are thus discerned by Skjærseth (2000, p. 35); while being ‘ambitious’ describes a country going over and beyond an agreement, ‘reluctant’ refers to only partial fulfillment, whereas an ‘intermediate’ approach implies being a loyal implementer. In addition parties can choose to be ‘indifferent’, with domestic measures unrelated to international commitments and possibly going in the wrong direction.

Falkner and Treib (2008) have proposed more vivid characterizations of the typical implementation patterns, featuring four ‘worlds of compliance’, ranging from a strong compliance culture with a *world of law observance*, to a *world of domestic politics* with aspirations to comply being overridden by domestic interest policy, to a *world of transposition neglect* or a *world of dead letters*, in which a kind of Potemkin scenery prevails, due to a ‘combination of politicized transposition and systematic shortcomings in enforcement and application’ (ibid.). While derived from implementation studies of EU directives, these categories complement and partly resemble those derived by Skjærseth (2000) from studies of wider international environmental agreements.

Differences between pioneers, leaders and laggards of environmental policy have previously been the focus of comparative studies of northern and southern Europe within the EU, although in recent years and in the context of climate policy also globally (Wurzel et al. 2020). Since a laggard is reluctant and resistant to the adoption of comprehensive and stringent environmental regulations, it means that a laggard state introduces certain policies comparatively late or not at all. In contrast, leaders and pioneers can act as agents of change (Liefferink and Wurzel 2017) who are of central importance for successful international action. While pioneering countries introduce policies and measures mainly for domestic reasons, to stimulate wider international action to address collective goods problems, leaders have the explicit aim of leading others, and if necessary, to push others to a follower position (ibid.). Nordic countries have long been considered to have performed as pioneers, and occasionally as leaders, in forging international environmental agreements (Andersen and Liefferink 1997). The post-Communist countries around the Baltic Sea have, despite aspirations as followers, a mixed reputation (Andersson 1999; Kontio and Kuitto 2013; Korppoo et al. 2015; Ptak et al. 2020). The Helsinki Convention thus offers a rather unique opportunity to study the patterns of domestic implementation within a common framework of actions agreed among a diverse set of countries.

As such it offers a micro-cosmos of the implementation and compliance challenges of a much wider set of international environmental agreements, including those relating to climate change, where leaders, laggards, followers and pioneers have reached agreement to join forces. Such agreements require unanimity and despite being legally binding under international law, they cannot be rigorously enforced, making domestic implementation ‘the moment of truth’ (Tynkkynen et al. 2014; Bohman 2018).

While the measures of the Convention mostly concern the actual modifications of production practices, e.g. farming technology that reduces applications or losses of nutrients or maximizes retention and denitrification, the Parties may choose to apply complementary policy instruments, motivating, pushing or enabling actors to do things they might not otherwise have done (Schneider and Ingram 1990). Policy instruments are conventionally grouped into the three categories of regulations, economic means and information, often characterized as *sticks*, *carrots or sermons* (Vedung 2011). In the case where economic means are used, the target group is not obliged to certain actions, which however can be facilitated or obstructed through the provision of or deprivation of financial resources. When using information the relationship is persuasive “involving only the communication of claims and reasons” (ibid., p. 48). The degree of constraint that is involved with a policy instrument reflects its ‘authoritative force’. Thus, in principle regulation is more constraining than economic means, and both are more constraining than the use of information.

How national governments combine the various policy instruments can be expected to differ, reflecting the degree of constraint that they wish to impose on target groups. The pattern of the policy instruments employed allows for a characterization of the stringency of the domestic implementation approaches of the various countries, which in turn allow us to classify them according to their level of ambition and mode of compliance with an international agreement.

## Materials and methods

Our research on the domestic implementation of Convention measures was carried out in a two stage process. Firstly, supported by national experts from each of the nine littoral states, the authors identified, mapped and analyzed current legislation and administrative practices, overcoming language challenges where present. For each country we tabulated the relevant domestic nutrient management measures, corresponding to the main elements of the Convention. Moreover, we identified estimates from the literature of the nutrient-reduction potential of the various measures to clarify their relative importance. Secondly, and to support the characterization of the domestic implementation, we conducted a literature search for relevant journal articles, books and research reports, including those from relevant European research programmes (e.g. BONUS and Interreg). We searched four main literature databases (‘Web of Science’ by ISI, Scopus, Google Scholar and Microsoft Academic) and used the tool ‘Publish or Perish’ by Harzing.com, which enables advanced searches in the Google Scholar and Microsoft Academic databases. We combined keywords (Baltic Sea, nitrogen, phosphorus, policy instruments and nutrient management measures) with country names and the year 2004 as the cut-off, which is about 5 years after adoption of the Annex as our interest is in subsequent implementation. These searches returned a large number of publications from which we selected those relevant to nutrient management practices prescribed by the Convention. Additional literature including some key older references were identified with snowballing techniques.

Moreover, four national and one pan-Baltic stakeholder workshops with participation from farmer groups, advisory services, ministries and NGO’s were organized as part of our research for clarifying uncertainties and supporting the mapping and characterizations (see Supplementary Material).

## Results

### Preexisting nutrient regulations of EU

As a framework for understanding the domestic implementation of the Convention requirements, we briefly revisit its precursor, the EU’s Nitrates Directive of 1991. It requires that Member States define ‘codes of good agricultural practices’. These codes are though not legally binding, and hence voluntary for farmers, except where Member States have identified so-called Nitrate Vulnerable Zones (NVZ); here action programs must be developed, whereby the behavioral codes become mandatory. Nowadays, several EU Member States have NVZ-designated their entire national territory, although of our littoral countries not Estonia, Latvia or Sweden (EC 2018).

According to the Nitrates Directive the code of good agricultural practices must identify embargo periods prohibiting applications of manure as well as the conditions for application on sloping grounds, near water courses or during periods of flooded or frozen ground. Moreover, the codes must specify the capacity requirements for storage of manure and the procedures for its spreading. Codes may also (optionally) prescribe the use of winter cover, crop rotations, fertilizer plans, nutrient book-keeping and other nutrient management measures. However, in NVZ-areas all of these measures become mandatory, complemented by further requirements, notably the ceiling of 170 kg N/ha for the spreading of manure. In NVZ-areas it is moreover mandatory to have storage capacity sufficient to match the longest embargo period during which spreading is prohibited, and there is a balancing requirement, stating that fertilizer use should not exceed nitrogen requirements of crops, while taking into account soil deposits and net mineralization of nitrogen.

In acknowledgement of the sensitive nature of the Baltic Sea, Convention requirements go further and apply to the entire national territory whether NVZ-designated or not. The Convention also widens the scope to phosphorus nutrients, and is more restrictive by, for instance, committing countries to set maximum densities for livestock and to specify a minimum of 6 months storage capacity for manure. The Convention explicitly commits countries to issue national guidelines or legislation on ten specified measures relating to nutrient management (Bohman 2018).

### Mapping of domestic implementation

We here review eight of the ten key measures (M1–10) of the Convention and the extent to which they have been implemented by the acceding countries.^3^ A country by country overview is shown in Table 1.

**Table 1:**
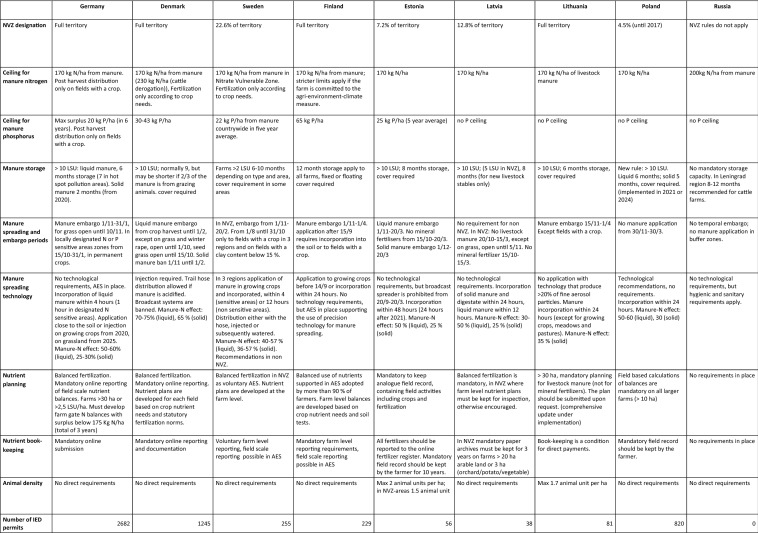
Measures for nutrient management

#### Manure storage

Besides requiring at least 6 months of storage capacity for manure (M3), the Convention prescribes that facilities for liquid manure (slurry) should have a cover (M10). Sufficient manure storage capacity is a critical measure because it supports optimal timing of manure spreading during the growing season of crops, thereby improving nutrient utilization efficiency (Jensen et al. 1994). Without adequate storage capacity, no more than about 20% of manure nitrogen will realistically be utilized by plants, whereas optimal storage capacity can support an uptake of up to 70% (Sørensen et al. 2017).

For slurry we find legal requirements for storage capacity of at least 6 months in all the countries, with the notable exception of Russia. Stricter standards apply in the Nordic countries (see Table 1), while storage capacity requirements for solid manure fall below standards in Germany and Poland. In fact, until recently Poland required only four months of storage capacity, even for slurry in NVZ-designated areas. Sarteel et al. (2016) estimate that in Poland 40–45% of manure is distributed in autumn when plant uptake is low and losses to the environment consequently high, and one survey finds that even among larger farms merely 12% have invested in manure storage facilities (Konrad et al. 2019). Everywhere but Sweden, storage capacity requirements apply only to farms with more than 10 Livestock Units (LSU), although the Convention has no such minimum threshold. In Poland and the Baltic countries 13–23% of all livestock is found on farms below 10 LSU (Eurostat 2020).^4^

We find legal requirements for covers to avoid ammonia evaporation only in some of the countries (Denmark, Estonia, Finland, Lithuania and NVZ-areas of Sweden). Germany has announced requirements for covers as from 2030. Our analysis is congruent with the findings of Rodhe et al. (2017) who report that 40–50% of all storage facilities in the region are not properly covered, while in the Baltic countries 10% of slurry is stored in open lagoons, implying high ammonia losses. Use of lagoons contradict Convention requirements for storage quality ‘to prevent losses’ and for ‘containers made of strong material impermeable to moisture’. Covering with roofing, plastic or floating cover (crust) reduces ammonia losses from storage by 80–90% (Loyon et al. 2016).

#### Manure spreading: Embargo periods and technology

Embargo periods for the application of manure in terms of bans on winter spreading (M6) are defined in all countries, except Russia. Mostly the embargo periods run from November 1st until February to March, but with numerous exemptions and special national clauses (see Table 1). Embargos are an effective way to prevent N losses as most leaching occurs during winter, when soils are water saturated or frozen and plant growth is minimal (Eriksen et al. 2014).

The Convention recommends that manure is incorporated directly after application on bare soil (M6), but has no specification of the spreading technologies to be used, except that manure ‘shall be spread in a way that minimizes the risk of loss of plant nutrients’ achieving a ‘high utilization efficiency’ (ibid.). Recent surveys among farmers show that simple broadcast spreading (into the air) is widely used (60–70%) in most countries, and permitted in existing national regulations (Rodhe et al. 2017; Konrad et al. 2019). Broadcast spreading implies losses of total-N that are 10–20% higher than spreading with trail hoses or injection (Jensen et al. 1994), and increase ammonia losses by 65% relative to the best injection technology (Kaasik 2012). Stringent requirements with a ban on broadcast spreading are defined only in Denmark and for Sweden’s NVZ-areas (Thorsøe et al. 2017). The convention requirements for rapid manure incorporation (within at least 24 h) are implemented by all Parties, except Russia, while stricter time limits apply in Germany and Sweden (see Table 1).

#### Ceilings for manure nutrients

We find that apart from Russia the nitrogen ceiling of 170 kg N/ha for manure nutrients (M7) has been transposed into national legislation by all Parties. However, three countries (Sweden, Estonia and Latvia) omit a ceiling in non-NVZ-areas. Russia maintains a higher national ceiling of 200 kg N/ha, while Denmark has obtained an EU derogation for cattle farms enabling 230 kg N/ha on about 10% of its agricultural land.

We find regulations in conformity with the P-ceiling of 25 kg P/ha (M7) only in Sweden and Estonia, whereas no ceiling is defined in Latvia, Lithuania, Russia or Poland. Recent P-limitations introduced in Denmark (30–43 kg P/ha), and Germany’s approach of allowing a surplus of 20 kg P/ha are both in contravention of the Convention requirements. Finland’s ceiling of 65 kg P/ha dramatically exceeds requirements, although farmers are offered voluntary payments for accepting stricter P-limits. Given the importance of achieving significant P-reductions, these deviations from the Convention requirements are surprising, especially for the countries with a high P-surplus per ha; Denmark, Finland and Russia (Svanbäck et al. 2019).

#### Animal densities

To avoid excessive production of animal nutrients, the Convention prescribes that countries should define a balance between the number of animals and the amount of land available for spreading manure, expressed as animal density (M1). It further stipulates that a ‘maximum number of animals should be determined’ while taking into account the balancing requirement, i.e. of crop requirements relative to the amount of nutrients applied. However, we find that restrictions on animal densities have been introduced only in Estonia and Lithuania, while Denmark revoked its previous requirements in 2017.

#### Winter crop cover

The Convention states that cultivated areas should be sufficiently covered by crops in autumn and winter to reduce nutrient losses in the ‘relevant regions’ (M8). We find that there are statutory requirements for winter cover crops in Denmark and in Sweden (nine regions) and Estonia (within NVZ). Winter cover crops are subject to very different rules as to what counts as winter cover, and how and when crops should be in place. The Nordic countries, Germany and Estonia use AES payments to support catch crops, though Sweden only in NVZ-areas. Catch crops account for respectively 5%, 8% and 10% of the agricultural area in Sweden, Denmark and Finland (Aronsson et al. 2016). Baltic countries and Poland have guidelines in place for what qualifies as winter cover crops, but no specific requirements or support schemes. Further, winter cover crops can qualify as ‘Ecological Focus Areas’ (EFA) that all farmers in EU countries must have to obtain the basic income support of the Common Agricultural Policy (CAP). Cover or catch crops (usually grasses) are effective for reducing N losses, with an uptake of 7 to 38 kg N/ha, depending on crops and soil types (Aronsson et al. 2016).

#### Water protection measures

The Convention requires that water protection measures, such as buffer zones and groundwater protection should be established ‘where necessary’ and urges countries to restore wetlands (M9). Buffer zones are mandatory for receiving the basic area support of the CAP, but designations of such zones differ according to the national codes of Good Agricultural Practices. While Sweden, Estonia and Poland have differentiated requirements ranging up to 20 m, requirements in Germany, Denmark and Finland are limited to 1–3 m, although permitted activities and waterbodies subject to buffer zones vary across countries (see Table 1). Buffer zones beyond this are voluntary and payments are offered to farmers from the schemes of the European Agricultural Fund for Rural Development (henceforth rural development). It is possible to count buffer zones as EFA’s, but the conversion factor differs among the countries. Groundwater protection zones as a policy instrument have a long tradition in Germany and was introduced recently in Denmark, but are not used much elsewhere. Buffer zones to protect water quality are particularly effective for P abatement (Liu et al. 2008), while a recent meta study finds N reductions of 33% in surface water runoff and 70% in groundwater, but much depends on width, age, management practice and nutrient concentrations (Valkama et al. 2019).

With respect to wetlands, we find that the Nordic countries have initiated comprehensive restoration programs (Graversgaard et al. 2021). Conversely, in Poland and the Baltic countries, where many natural wetlands have been retained, development of farmland drainage is a priority for financial support from rural development funds. Although nutrient removal rates of wetlands vary, a recent systematic review indicates rates corresponding to 37–46% of inflows (Land et al. 2016).

#### Permits

The Convention requires that large livestock farms (> 400 LU) be treated as point sources and have an integrated environmental permit covering emissions to all media, based on the principle of Best Available Technology (BAT). For EU Member States, such permits are required under the Industrial Emissions Directive (IED) for large-scale livestock facilities, ensuring that BAT is implemented and that frequent controls are carried out. Still, the number of facilities with permits varies significantly among the countries (see Table 1) and permit requirements are not fully harmonized; notably with regard to manure spreading (Kauppila and Anker 2018). Moreover Loyon et al. (2016) observe that the IED list of BAT is incomplete, missing various manure treatment options, and needs regular updates to keep pace with technological developments.

### Complementing sermons and carrots

We here further review the sermons and carrots that the Parties have instituted to complement their implementation of the regulations (sticks) following from the Convention, and their relative importance. A country by country overview is shown in Table 2.

**Table 2:**
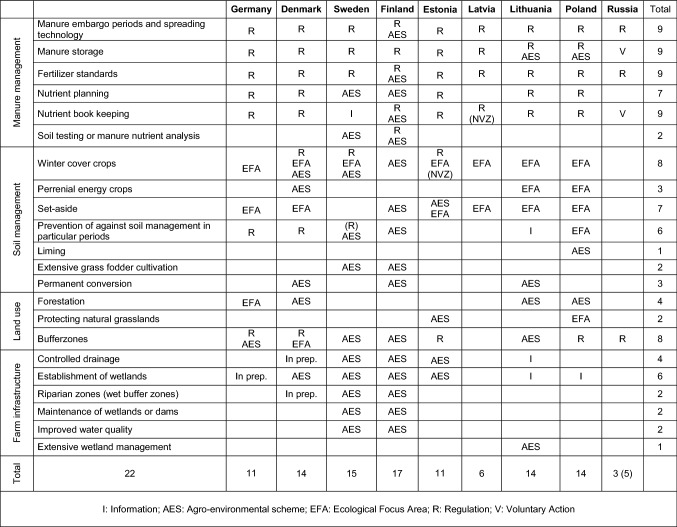
Policy instruments for nutrient management

Sweden has for many years operated a national farmer advisory program “Catch the Nutrients” (*Greppa Näringen*), where extension services help improve nutrient management and lower nutrient surpluses, while improving farm profitability (Nordin and Höjgård 2017). In Finland, some regional projects have offered similar farm specific advice (Launto-Tiuttu et al. 2014). Pilot experiments conducted in five countries suggest that allocating sufficient resources for stakeholder involvement is critical for effective nutrient management (Neset et al. 2019). However, capacity building involving training and outreach activities like demonstration projects, direct technical assistance, newsletters and seminars is mostly ad hoc, and is not pursued systematically in those countries where it is most needed. Following case studies in Estonia, Latvia, Poland and Germany, Fammler et al. (2018) observe that most farmers do not understand the need for nutrient management, as they are not well aware of the effects of their fertilizer practices. Ensuring targeted support and capacity building is therefore a key challenge for policy makers that has not been sufficiently addressed (Taylor et al. 2012). Daberkow et al. (2008) identify three preconditions for training and education alone to be effective for reducing emissions, but that rarely converge (1) opportunities for a win–win scenario improving both environment and farm profitability, (2) producers with strong altruistic motives and (3) high private costs of environmental degradation.

All EU countries have according to the requirements of the Nitrates Directive developed codes of good agricultural practices. However, the codes everywhere but Denmark abstain from providing explicit values for the nutrient contents of manure as needed to substitute mineral fertilizer (Webb et al. 2013). Such information can only be found elsewhere, e.g. in reports or in non-binding administrative circulars, providing optional values at a modest level of substitution (Webb et al. 2013; Laakso and Luostarinen 2019).

From 2013, 30% of the CAP’s direct income support has been made contingent on ‘greening’ elements, under which mandatory EFA’s covering at least 5% of the farmland are required. EFA’s can be implemented in various ways, for instance as cover crops, buffer zones or set-aside. Moreover, Agri-Environmental Schemes (AES) are optional instruments, where farmers are financially compensated for adopting practices that reduce nutrient losses (Gunningham and Sinclair 2005). The national level Rural Development Programs (RDP) can be used to provide financial support for AES that reduce nutrient losses, e.g. extended buffer zones. Thus there are ample opportunities for underpinning nutrient management with financial resources from the EU, although RDP support requires national co-funding. Still, it is optional for member states whether to actually make use of RDP funds for reducing nutrient losses or not.

We have mapped the AES relevant to nutrient management, involving schemes for buffer zones, catch crops, set-aside and forestation. While buffer zones are mandatory in Estonia, Russia and Poland they are voluntary and encouraged with AES in Sweden, Finland and Lithuania. Denmark combines the two approaches as 2 m of buffer zones are mandatory and an additional 1–20 m may be implemented to meet EFA requirements or as an alternative to mandatory catch crops. Furthermore, intermediate crops (catch crops with short rotation) can be used both as AES with payment and to meet EFA requirements. AES for mandatory investment in manure storage was used in the early phase in Denmark and in recent years to a limited extent in Poland and Lithuania. Denmark, Sweden and Finland use AES to support the creation or restoration of wetlands. Estonia, Latvia, Lithuania and Poland have no AES for maintaining or creating wetlands, and in general have made very limited use of RDP Funds for nutrient management (Kociszewski 2013).

## Characterizations of domestic implementation patterns

It is evident that a large number of Convention measures have been implemented in most countries, though considerable differences prevail.

Six measures must be considered mandatory according to the Convention: capacity for 6 months manure storage, embargo periods for manure spreading, per hectare ceilings for manure N and P application, respectively, covers on manure slurry storage facilities and permits for large livestock farms. Three measures without specified requirement levels should rather be considered optional: winter cover crops, buffer zones and wetlands.

With regard to embargo periods all countries except Russia have defined such periods, however in defining adequate storage requirements not only Russia, but also Poland and Germany are not in compliance. These three countries are joined by Latvia in not having requirements for storage covers in place. Finally, Denmark, Finland and Lithuania along with the previous five are in contravention of the ceiling for manure P-applications. This leaves Sweden and Estonia as the most compliant countries with respect to the Convention measures. Still, their compliance fade in non-NVZ-designated areas, corresponding to about 40% and 66% of the utilized agricultural area for Sweden and Estonia, respectively.

With regard to the optional measures, the patterns are more complex, as their implementation are tied in with the frameworks of payments to farmers. Cover crops, buffer zones and wetlands are an option in the eight EU Member States, but the extent to which countries offer payments from the available EU funds differs. Since EFA’s are mandatory for receiving full CAP income support payments, all countries have some mechanisms in place that allow for flexibility and conversion of EFA’s to other land-use measures such as catch crops or buffer zones. However, with regard to RDP funds, only Sweden, Finland and Denmark offer payments for the three optional Convention measures. Estonia, Latvia, Lithuania, Poland and Germany have abstained from making use of this opportunity when setting RDP priorities.

Russia is a special case as the only country not in the EU. Its regulatory approach emphasizes sanitary and hygienic standards of water quality, and contains no explicit requirements for agricultural nutrient management, apart from designation of buffer zones and a 200 kg N/ha ceiling. Some projects funded by HELCOM and bilaterally have targeted large livestock facilities, but funding from the Russian Federation itself has despite pledges not been allocated (Tynkkynen 2018). Eutrophication has always been a low priority for Russia and the risks to human health from nitrate pollution of air and water are not acknowledged, explaining its neglect and laggard behavior on nutrients (Korppoo et al. 2015).

Poland covers 48% of the Convention Parties’ farmland, but has for many years been reluctant to transpose the agreed measures into national legislation, limiting manure storage requirements to 4 months for example. While spending merely 4% of RDP funds for AES (Kociszewski 2013), Poland until 2018 had NVZ-designated only 4.5% of its territory. Despite the ruling by the European Court (C-356/13) on Poland’s lax implementation of the Nitrates Directive (cf. Kowalczewska et al. 2018), which triggered NVZ designation of the entire territory, we did not find any evidence of changes in the actual policy instrument mix, which continues to rely on regulations and offers limited economic funding for AES (Szalińska et al. 2018).

We observe comparable patterns in Estonia, Latvia, Lithuania and Germany with respect to the use of policy instruments—an emphasis on sticks with hardly any optional payments being provided, though these countries have done more to implement Convention measures than Poland. Still, Germany has been foot-dragging in following-up on the judgement from the European Court (C-543/16) on its poor implementation of the Nitrates Directive, triggering new infringement procedures.

Among the Nordic countries, Denmark has, despite some critical shortcomings, nevertheless the broadest array of measures and policy instruments in place, including some that go beyond Convention requirements, but it has also the highest livestock concentrations in the region. A strict management regime was instituted in the 1990s with numerous regulations, e.g. mandatory nutrient utilization requirements, fertilizer planning and bookkeeping adapted to large-scale agricultural production (cf. Andersen et al., 2014). In contrast Sweden and Finland have a mix of policy instruments that emphasize AES payments and information, with less stringent requirements, especially in Finland (Marttinen et al. 2018). Land-use measures such as constructed wetlands are more widely used by Finland too, while Sweden offers free nutrient advisory services, aiming to complement regulations by encouraging voluntary reductions beyond these. Denmark focuses more on soil management measures like catch crops.

The different approaches are reflected in the allocation of CAP funds. Sweden, Finland, Estonia, Latvia and Lithuania allocate high shares (30–50%) for their RDP, while Germany, Poland and Denmark allocate more funding for direct income support (80–90%) (own calculation based on EP 2020). Allocating funds for RDP implies more administrative and political control on the spending as well as a need for co-financing, but the extent to which it has been used to the benefit of nutrient management is not proportional to the shares allocated. Sweden uses just 1.6% of total CAP support for voluntary payments for nutrient management (cf. SCB 2018: Table 9.1). CAP support is overall less generous in Estonia, Lithuania, Poland and especially Latvia, with RDP used mainly for modernizations and improving agricultural competitiveness, reflecting that economic development has a higher priority than nutrient management (cf. ECA 2016). Payments in Russia stem largely from foreign donors.

Codes for good agricultural and environmental practices, as required under the EU’s Nitrates Directive, offers a further policy instrument for promoting efforts among farmers. While all the EU countries are using this policy instrument, in practice, the codes fall short of providing guidance on the nutrient contents of manure and how it is best used to substitute for mineral fertilizers. This is unfortunate as many farm workers lack basic knowledge on these aspects, especially in the post-Communist countries (BalticDeal 2011; Drangert et al. 2017).

## Discussion

Our findings show that implementation has evolved in a rather piecemeal way across the countries of the region, despite the optimism expressed 10 years ago about the prospects for successful followership, i.e. uptake of the prescribed agricultural measures (Hjorth 1998; Roginko 1998). In speculating about why the post-Communist countries in the first place were willing to sign up to rather detailed and prescriptive measures, it seems evident that the specific geopolitical circumstances of the year 1998 must have played a key role. Poland and Estonia were in accession negotiations, having received invitations for membership of the EU, while Latvia and Lithuania were candidate countries striving towards the same goal. For this they needed the support of the European Commission, which held the presidency of HELCOM, as well as of Germany and the Nordic countries and were presumably willing to go a long way to build a good relationship, sacrificing concerns over the possible costs to farmers in the conviction that EU membership would bring access to financing and funds. To Russia, 1998 was the ultimate downturn, with the government’s bankruptcy following the turmoil of transforming into a market economy. Consequently, the 1998 amendment to the Convention must have been an issue of minor concern to Russia. Nevertheless, with the disintegration into a loose confederation under President Yeltsin’s faltering leadership, it conveniently reconfirmed Russia’s role in the Baltic Sea region, while leveraging transfer of support funds from the west. Following the privatization of former collective farms and the termination of Soviet subsidized fertilizer use, agriculture had collapsed in Estonia, Latvia, Lithuania and northwest Russia, diminishing the possible nutrient losses greatly. These circumstances help explain why for the post-Communist countries it was difficult, if not impossible, to gauge the implications of the measures agreed to, thus creating a window of opportunity for the EU and the lead countries Denmark and Sweden to add agricultural regulations to the Convention, inspired by approaches pioneered by them.

Still, our analysis of domestic implementation has unveiled somewhat surprising patterns in that three out of four of the old EU Member States are not respecting fully the provisions of the Convention relating to agricultural nutrients. While Germany’s lax implementation is very much in line with a *World of domestic politics*, both Finland and Denmark are not quite up to the *World of law observance* as predicted by Falkner and Treib (2008) for Nordic countries. They are rather somewhere between these two worlds. Nor are Poland, Latvia and Lithuania found to be firmly in the *World of dead letters* as predicted for new EU Member States, as we could observe partial implementation efforts, corresponding perhaps rather to a *World of symbolic action*. Still, Russia convincingly fits the theoretical categorizations, displaying a *World of transposition neglect*, as does Sweden, behaving in accordance with the *World of law observance*, somewhat surprisingly followed by Estonia.

As a result of these patterns the policy instruments are sometimes applied differently than according to their degree of authoritative force; notably where sticks tend to be symbolic they are hardly complemented by sermons or carrots.

Henkin (1968) observes that although violations of international agreements are generally rare, they tend to occur where the advantages of non-compliance overshadow the possible benefits of the agreement or where there are strong pressures from domestic interest groups who would benefit from lax implementation. Governments moreover do not always act on a careful calculus of cost and advantage, sometimes violation is unintentional or committed by other entities than those responsible for forging international agreement. Skjærseth (2000, p. 42) thus observes that “governments defect not so much by deliberate choice, but rather owing to lack of implementation *ability* due to resistance at sub-national level”. In our case, the responsibility for implementation of the Convention measures rests with the national Ministries of Agriculture or equivalent, that have generally been facing other priorities and challenges. As developing RDP’s are their responsibility, it explains why these financial resources have hardly been mobilized, especially in the post-Communist EU countries where the need for national co-funding is a further impediment. We consider these governance issues in more detail elsewhere (Andersen et al. 2021).

As smaller countries in the longer run have more benefit from the respect of international agreements, it is hardly surprising that we observe somewhat higher compliance and one clear example of followership among them. As to the larger countries, Germany, Poland and Russia, their Exclusive Economic Zones (EEZ) cover less than 20% of the Baltic Sea, and are bordering peripheral regions away from their capitals. Sweden with an EEZ of 33% and Finland with 21% stand to benefit the most from a cleaner marine environment, as confirmed also in surveys (see Ahtiainen et al. 2014). Correspondingly, Estonia trumps Latvia and Lithuania on coastline and EEZ, as well as Denmark, situated at the outlet of the Sea.

As trends in actual water quality indicators are difficult to interpret, we close with a tentative benchmarking of domestic implementation outcomes by considering the nutrient surpluses, reflecting the national differences between inputs of nutrients and outputs in agricultural products (see Table 3). The surpluses indicate trends over time in the problem pressure and suggest how well countries have managed to control farmers, although influenced also by structural and market developments.

**Table 3:** Average annual nutrient surplus per unit of farmland

	1997–2003 (kg N/ha)	2015–2017 (kg N/ha)	Change (kg N/ha)	Change (%)	1997–2003 (kg P/ha)	2015–2017 (kg P/ha)	Change (kg P/ha)	Change (%)
DK	127	^¤^80	− 47	− 37	13.1	^¤^7.0	− 6.1	− 47
DE^a^	103	70	− 33	− 32	3.1	− 3.3	− 6.5	− 206
EE	^¤^36	^¤^22	− 14	− 39	− 5.0	^¤^− 7.0	− 2.0	− 40
FI	61	49	− 12	− 20	9.3	4.7	− 4.6	− 50
LV	14	25	+ 11	+ 80	0.4	1.3	+ 0.9	+ 211
LT	34	^¤^25	− 9	− 27	5.5	^¤^1.0	− 4.5	− 82
PL	43	47	+ 4	+ 8	3.7	1.5	− 2.2	− 60
RU^b^	144	^¤^130	− 14	− 9	10.5	^¤^16.5	+ 6.0	+ 57
SE	52	35	− 17	− 33	2.3	0.7	− 1.6	− 71

1997–2003 is BSAP baseline

^a^DE: national

^b^RU: Baltic Sea catchment; ^¤^EE: base year 2004; DK, EE, LT: 2015 data only; RU: no 2017 data

*Sources* Eurostat and own calculations based on Russia’s Federal State Statistics Service by Knoema.com

Russia clearly tops the surplus rankings, owing to declines in agricultural areas and more livestock, while surpluses in Estonia, Latvia and Lithuania are at a low level. In terms of trends over time from the baseline years to the most recent data published, large reductions in nutrient surpluses in Denmark and Germany^5^ are notable, reflecting changes in farmer behavior. The N surplus figures moreover provide indications of substantial behavioral change in Sweden and Estonia, suggesting their stringent measures are having effect. Despite the main emphasis across the Baltic Sea region having been on managing agricultural N (Liu et al. 2018), the P surplus has also declined in all countries (but Latvia and Russia) and at a higher rate than for N, which is explained by the doubling of the world market price for raw phosphorus during the period.^6^

While it would require detailed data and econometric techniques to disentangle the reductions achieved with specific measures and policy instruments, we observe that the N surplus trends are broadly in line with what we might hypothesize based on the above analysis: limited reductions if any in Poland and Russia, and in relative terms notable achievements in Sweden and Estonia. Changes are evident in Germany and Denmark too, but both countries maintain a high absolute surplus, reflecting their intensive livestock production. In Finland and Lithuania the N surplus changes are modest. Indeed it is in the Gulf of Finland, Riga Bay and the Baltic proper that reductions are most needed (HELCOM 2018a).

When the initial Convention measures were agreed in 1998, the data on nutrient losses were still patchy and the scientific understanding of the basic biophysical relations in an emerging phase. The question is whether the recent interest among policy makers to improve cost-effectiveness by tailoring regulations better to the biophysical evidence base on nutrient pathways (cf. OECD 2018) implies, that the Convention approach of specifying measures is becoming somewhat obsolete. However, it is difficult to see how the minimum measures identified by the Convention of storage capacity, suitable covering, embargo periods and maximum ceilings for application should not remain relevant. They represent a significant joint commitment to the basics, beyond which countries can opt for more targeted ways to control nutrients, e.g. addressing farmlands prone to high leaching rates, or by making financial contributions across the catchment to support low-cost reductions (see Andersson et al. 2021).

## Conclusion

In this article, we have analyzed the patterns of domestic implementation of the measures agreed to under the Helsinki Convention on the Protection of the Marine Environment of the Baltic Sea Area for managing nutrient losses from agriculture in the nine littoral states. We find that all countries, with the notable exception of Russia, have implemented several of the agreed measures. However, we also identify major shortcomings in virtually all countries; Poland and Germany have inadequate rules on regulation of storage capacity for manure, and they are joined by Latvia in not requiring permanent covers on storage containers to limit ammonia evaporation. These countries jointly with Denmark, Finland and Lithuania have not implemented the phosphorus-application ceiling. Sweden and Estonia have the highest level of compliance, though only on part of their territory. Moreover, guidelines on maximum animal densities are missing in all the countries with large livestock concentrations. Where countries are using payments to farmers as a policy instrument for promoting the implementation of nutrient management measures, these are predominantly sourced from the EU. There is limited, if any, national funding offered to compensate for the lack of authoritative enforcement of the agreed measures.

Our mapping thus unveils a somewhat patchy implementation of the international environmental agreement to protect the Baltic Sea. The degree to which countries are violating the agreement seems partly related to the inability of gauging appropriately the advantages and burdens that it involves for them, at the time of concluding the agreement. The post-Communist countries in particular faced difficulties in this respect, and while receiving financial support, they have been able to divert them for their own domestic purposes. We see the underperformance on or neglect of concluded agreements to reflect not only that most countries are vulnerable to domestic politics overriding their international commitments, but also that such commitments have been accepted in settings where much larger geopolitical and security interests were at stake.

The risk of arriving in a *World of transposition neglect*, or in a *World of dead letters* that stalls the domestic implementation of an international agreement has relevance far beyond the Helsinki Convention. We see in the climate negotiations how developing countries and emerging economies are willing to go a long way to accept demanding reduction targets on the condition of financial and technological transfers from the countries that are pushing for action, and how larger geopolitical considerations influence the building of alliances in this respect. An appropriate response to such risks is no doubt to build stronger international institutions to oversee and guard the agreements made. With the EU as a signatory to the Helsinki Convention there seems to be a missed opportunity to gain legal traction for the agricultural measures agreed, with the river basin management plans that are compulsory for member states under the EU Water Framework Directive (see Brady et al. 2021). Looking beyond the EU, to obtain compliance from Russia will be no small challenge either, and is likely to become a crucial issue in the context of other important international environmental agreements. The ambiguous experience gained from efforts to control agricultural pollution of the Baltic Sea should spur further analysis of how to strengthen countries’ commitments to supranational agreements.

## Supplementary Information

Below is the link to the electronic supplementary material.

Supplementary file1 (PDF 696 kb)

## Footnotes

1 https://helcom.fi/media/publishingimages/Helsinki-Convention_July-2014.pdf.

2 “The Contracting Parties shall integrate the following basic principles into national legislation or guidelines and adapt them to the prevailing conditions within the country to reduce the adverse environmental effects of agriculture. Specified requirement levels shall be considered to be a minimum basis for national legislation.” See part 2 of https://helcom.fi/about-us/convention/annexes-to-the-convention-2/annex-iii/.

3 We focus on the main sources and most well defined measures, thus omitting ‘location and design of animal houses’ (M2) and ‘agricultural wastewater and silage effluent’ (M5).

4 The annual volume of manure from 10 dairy cows (= 10 LSU) is about 310 metric tonnes containing approximately 1.3 tN and 0.2 tP, cf. Tybirk et al. (2013).

5 No time series of the gross nutrient surpluses of Germany’s Baltic Sea catchment are available, but in absolute terms they seem fairly close to the national average, see 2. Nährstoffbericht des Landes Schleswig–Holstein (2020) and Mecklenburg-Vorpommern Regionalisierte Nährstoffbilanzen landwirtschaftlicher Nutzflächen (2013).

6 https://www.indexmundi.com/commodities/?commodity=rock-phosphate&months=240.

## References

[CR1] Ahtiainen H, Artell J, Czajkowski M, Hasler B, Hasselström L, Huhtala A, Meyerhoff J, Smart JCR (2014). Benefits of meeting nutrient reduction targets for the Baltic Sea. Journal of Environmental Economics and Policy.

[CR2] Andersen HE, Blicher-Mathiesen G, Bechmann M, Povilaitis A, Iital A, Lagzdins A, Kyllmar K (2014). Mitigating diffuse nitrogen losses in the Nordic-Baltic countries. Agriculture, Ecosystems and Environment.

[CR3] Andersen MS, Liefferink D (1997). European environmental policy: The pioneers.

[CR4] Andersen MS, Andersson A, Brady MV, Graversgaard M, Kilis E, Pedersen AB, Thorsøe M, Valve H (2021). Agricultural nutrient governance and implementation of international commitments: How domestic institutions matter, BONUS TOOLS2SEA.

[CR5] Andersson A, Brady MV, Pohjola J (2021). Unnecessarily high abatement costs and unfair distribution of costs hinder to Baltic Sea action on nutrient emissions—According to a synthesis of the literature. BONUS TOOLS2SEA.

[CR6] Andersson M (1999). Change and continuity in Poland’s environmental policy.

[CR7] Aronsson H, Hansen EM, Thomsen IK, Liu J, Øgaard A, Känkänen H, Ulén B (2016). The ability of cover crops to reduce nitrogen and phosphorus losses from arable land in southern Scandinavia and Finland. Journal of Soil and Water Conservation.

[CR8] BalticDeal. 2011. *Agri-environmental measures in the Baltic Sea Region*. www.balticdeal.eu. Accessed 21 Jan 2013.

[CR9] Bartosova A, Capell R, Olesen JE, Jabloun M, Refsgaard JC, Donnelly C, Hyytiäinen K, Pihlainen S (2019). Future socioeconomic conditions may have a larger impact than climate change on nutrient loads to the Baltic Sea. Ambio.

[CR10] Bohman, B. 2017. Transboundary law for social–ecological resilience. Doctoral Dissertation, Stockholm University.

[CR80] Bohman, B. 2018. Lessons from the regulatory approaches to combat eutrophication in the Baltic Sea region. *Marine Policy* 98: 227–236.

[CR11] Brady MV, Andersen MS, Andersson A, Kilis E, Sareela S-R, Thorsøe M (2021). Strengthening the policy framework to resolve lax implementation of the Baltic Sea Action Plan for agriculture. Ambio.

[CR12] Daberkow, S., M. Ribaudo, and O. Doering. 2008. Economic implications of public policies to change agricultural nitrogen use and management. In *Nitrogen in agricultural systems*, eds. J.S. Schepers and W.P. Raun, 883–910. Wiley Online Library.

[CR13] Dalgaard T, Hansen B, Hasler B, Hertel O, Hutchings NJ, Jacobsen BH, Jensen LS, Kronvang B (2014). Policies for agricultural nitrogen management. Environmental Research Letters.

[CR14] Drangert J, Kiełbasa B, Ulen B, Tonderski KS, Tonderski A (2017). Generating applicable environmental knowledge among farmers. Agroecology and Sustainable Food Systems.

[CR15] EC (2018). Report from the Commission to the Council and the European Parliament on the implementation of Council Directive 91/676/EEC.

[CR16] ECA (2016). Combating eutrophication in the Baltic Sea: Further and more effective action needed.

[CR17] EMEP (2013). Atmospheric supply of nitrogen, lead, cadmium, mercury and dioxins/furans to the Baltic Sea in 2013. EMEP/MSC-W Technical Report 2.

[CR18] EP. 2020. *The Common Agricultural Policy in figures (Table V)*. Brussels: European Parliament. https://www.europarl.europa.eu/factsheets/en/sheet/104/bendra-zemes-ukio-politika-isreiksta-skaiciais. Accessed 17 Dec 2020.

[CR19] Eriksen, J., P.N. Jensen, and B.H. Jacobsen. 2014. *Policy instruments to accomplish second generation river basin management planning and targeted land use management*. Aarhus University: DCA-Nationalt Center for Fødevarer og Jordbrug (in Danish). http://pure.au.dk/portal/files/84646400/Virkemiddelkatalog_web.pdf. Accessed 8 Dec 2017.

[CR20] Eurostat. 2020. *Main livestock indicators by NUTS 2 regions*. https://ec.europa.eu/eurostat/en/web/products-datasets/-/EF_LSK_MAIN. Accessed 2 April 2020.

[CR21] Falkner G, Treib O (2008). Three worlds of compliance—Or four? The EU15 compared to new Member States. Journal of Common Market Studies.

[CR22] Fammler, H., H.S. Weber, T. Fawzy, M. Kuris, L. Remmelgas, K. Veidemane, T. Bryan, K.S. Johansen, et al. 2018. The story of eutrophication and agriculture of the Baltic Sea. https://www.responseable.eu/wp-content/uploads/key-story-eutrophication-0518.pdf. Accessed 2 April 2020.

[CR23] Graversgaard M, Dalgaard T, Odgaard MV, Hoffmann CC, Jacobsen BH, Kjaergaard C, Powell N, Strand JA (2021). Policies for wetlands implementation in Denmark and Sweden. Land Use Policy.

[CR24] HELCOM (2018). Sources and pathways of nutrients to the Baltic Sea. Baltic Sea Environment Proceedings 153.

[CR25] HELCOM (2018). State of the Baltic Sea—Second HELCOM holistic assessment 2011–2016. Baltic Sea Environment Proceedings 155.

[CR26] Henkin L (1968). How nations behave.

[CR27] Hjorth R, Victor D, Raustiala K, Skolnikoff EB (1998). Implementation of Baltic Sea pollution commitments in Poland. The implementation and effectiveness of international environmental commitments.

[CR28] Jensen K, Høy JJ, Knudsen L, Maegård E, Andersen MS (1994). The impact of application equipment on the utilisation of manure. Spredningen af renere teknologi i landbruget, *Arbejdsrapport 59*.

[CR29] Kaasik A, Jakobsson C (2012). Chapter 16: Techniques for application of manure to land. Sustainable agriculture.

[CR30] Kauppila J, Anker HT (2018). The role of permits in regulating livestock installations and manure spreading. European Energy and Environmental Law Review.

[CR31] Kociszewski K (2013). Common agricultural policy instruments as factors of environmental sustainable development of Polish agriculture. Economic Environmental Studies.

[CR32] Konrad MT, Nielsen HØ, Pedersen AB, Elofsson K (2019). Drivers of farmers' investments in nutrient abatement technologies in five Baltic Sea countries. Ecological Economics.

[CR33] Kontio P, Kuitto K, Joas M, Jahn D, Kern K (2013). Environmental governance in the Baltic States. Governing a common sea: Environmental policies in the Baltic Sea Region.

[CR34] Korppoo A, Tynkkynen N, Hønneland G (2015). Russia and the politics of international environmental regimes.

[CR35] Kowalczewska K, Behagel J, Turnhout E (2018). Infrastructures of expertise: Policy convergence and the implementation of the EU Nitrates Directive in Poland. Journal of Environmental Planning and Management.

[CR36] Kremser U (1997). Agriculture within the context of HELCOM's mandate and activities (The Royal Colloquium. The Baltic Sea Region: Agriculture and Sustainability). Ambio.

[CR37] Laakso, J., and S. Luostarinen. 2019. Legislation and voluntary actions regulating manure fertilization and fertilizer use in the Baltic Sea Region. https://www.luke.fi/manurestandards/en/how-manure-fertilization-and-fertilizer-use-are-regulated-in-baltic-sea-region. Accessed 17 Dec 2020.

[CR38] Land M, Granéli W, Grimvall A, Hoffmann CC, Mitsch WJ, Tonderski KS, Verhoeven JTA (2016). How effective are created or restored freshwater wetlands for nitrogen and phosphorus removal? A systematic review. Environmental Evidence.

[CR39] Launto-Tiuttu, A., J. Heikkinen, J. Koskinen, E. Lankinen, E. Lundström, S. Puustinen, J. Röytiö, E. Vartiainen, et al. 2014. Targeted measures bring the greatest benefits for environmental protection in agriculture. *TEHO Plus Project.* https://www.doria.fi/handle/10024/102392. Accessed 17 Dec 2020.

[CR40] Liefferink D, Wurzel R (2017). Environmental leaders and pioneers. Journal of European Public Policy.

[CR41] Liu J, Kleinman PJA, Aronsson H, Flaten D, McDowell RW, Bechmann M, Beegle DB, Robinson TP (2018). A review of regulations and guidelines related to winter manure application. Ambio.

[CR42] Loyon L, Burton CH, Misselbrook T, Webb J, Philippe FX, Aguilar M, Doreau M, Hassouna M (2016). Best available technology for European livestock farms. Journal of Environmental Management.

[CR43] Marttinen, S., O. Venelampi, A. Iho, K. Koikkalainen, E. Lehtonen, S. Luostarinen, K. Rasa, M. Sarvi, et al. 2018. Towards a breakthrough in nutrient recycling. https://jukuri.luke.fi/handle/10024/542012. Accessed 17 Dec 2020.

[CR44] Neset T, Wilk J, Navarra C, Capell R, Bartosova AJA (2019). Visualization-supported dialogues in the Baltic Sea Region. Ambio.

[CR45] Nordin M, Höjgård S (2017). An evaluation of extension services in Sweden. Agricultural Economics.

[CR46] OECD (2018). Human acceleration of the nitrogen cycle.

[CR47] Ptak EN, Graversgaard M, Refsgaard JC, Dalgaard T (2020). Nitrate management discourses in Poland and Denmark. Water.

[CR48] Rodhe, L., J. Casimir, and E. Sindhöj. 2017. *Possibilities and bottlenecks for implementing slurry acidification techniques in the Baltic Sea Region*. RISE Research Institutes of Sweden. http://balticslurry.eu/wp-content/uploads/2016/06/Report-2.1-Possibilities-and-bottlenecks-REVISED.pdf. Accessed 17 Dec 2020.

[CR49] Roginko A, Victor D, Raustiala K, Skolnikoff EB (1998). Domestic implementation of Baltic Sea pollution commitments in Russia and the Baltic states. The implementation and effectiveness of international environmental commitments.

[CR50] Sarteel, M., C. Tostivint, A. Landowski, C. Basset, K. Muehmel, S. Lockwood, H. Ding, N. Oudet, et al. 2016. *Resource efficiency in practice: Closing mineral cycles*. Brussels: European Commission. https://ec.europa.eu/environment/water/water-nitrates/pdf/Closing_mineral_cycles_final%20report.pdf. Accessed 17 Dec 2020.

[CR51] SCB (2018). Agricultural statistics.

[CR52] Schneider A, Ingram H (1990). Behavioral assumptions of policy tools. The Journal of Politics.

[CR53] Skjærseth JB (2000). North Sea Cooperation: Linking international and domestic pollution control.

[CR54] Svanbäck A, McCrackin ML, Swaney DP, Linefur H, Gustafsson BG, Howarth RW, Humborg C (2019). Reducing agricultural nutrient surpluses in a large catchment. Science of the Total Environment.

[CR55] Szalińska E, Orlińska-Woźniak P, Wilk P (2018). Nitrate vulnerable zones revision in Poland. Sustainability.

[CR56] Sørensen P, Thomsen IK, Schröder JJ (2017). Empirical model for mineralisation of manure nitrogen in soil. Soil Research.

[CR57] Taylor C, Pollard S, Rocks S, Angus A (2012). Selecting policy instruments for better environmental regulation. Environmental Policy and Governance.

[CR58] Thorsøe, M., T. Dalgaard, and M. Graversgaard. 2017. *Comparative assessment of nitrogen and phosphorus policy instruments*. Aarhus University: DCA-Nationalt Center for Fødevarer og Jordbrug (in Danish). http://pure.au.dk/portal/files/117207724/DCArapport104.pdf. Accessed 21 Nov 2018.

[CR59] Tybirk, K., S. Luostarinen, L. Hamelin, L. Rodhe, S. Haneklaus, H. Poulsen, and A. Jensen. 2013. Sustainable manure management in the Baltic Sea Region. https://jukuri.luke.fi/bitstream/handle/10024/481921/sustmanure.pdf?sequence=1. Accessed 14 April 2020.

[CR60] Tynkkynen N (2018). The “Russian Issue” in transnational governance of the Baltic Sea environment. Marine Policy.

[CR61] Tynkkynen N, Schönach P, Pihlajamäki M, Nechiporuk D (2014). The governance of the mitigation of the Baltic Sea eutrophication. Ambio.

[CR62] Valkama E, Usva K, Saarinen M, Uusi-Kämppä J (2019). A meta-analysis on nitrogen retention by buffer zones. Journal of Environmental Quality.

[CR63] Vedung E, Bemelmans-Videc M, Rist RC, Vedung E (2011). Policy instruments: Typologies and theories. Carrots, sticks, and sermons: Policy instruments and their evaluation.

[CR64] Webb J, Sørensen P, Velthof G, Amon B, Pinto M, Rodhe L, Salomon E, Hutchings N (2013). An assessment of the variation of manure nitrogen efficiency throughout Europe and an appraisal of means to increase manure-N Efficiency. Advances in Agronomy.

[CR65] Wurzel R, Andersen MS, Tobin P (2020). Climate governance across the globe. Pioneers, leaders and followers.

[CR66] Øygarden L, Deelstra J, Lagzdins A, Bechmann M, Greipsland I, Kyllmar K, Povilaitis A, Iital A (2014). Climate change and the potential effects on runoff and nitrogen losses in the Nordic-Baltic region. Agriculture, Ecosystems and Environment.

