# Supplementary material for: Promise and performance of agricultural nutrient management policy: Lessons from the Baltic Sea
Source: Ambio. 2021 May 27;51(1):36–50. doi: 10.1007/s13280-021-01549-3 (PMC8651915; doi:10.1007/s13280-021-01549-3)
Supplement: Supplementary file 1 — Supplementary file1 (PDF 696 kb) [file 13280_2021_1549_MOESM1_ESM.pdf]

Promise and performance of agricultural nutrient management policy: lessons from the Baltic Sea

Martin H. Thorsøe[a]; Mikael Skou Andersen[b;] Mark V. Brady[c]; Morten Graversgaard[a] ; Emils Kilis[d]; Anders Branth Pedersen[b]; Samuli Pitzén[e]; Helena Valve[e];

[a]Dept. of Agroecology, Aarhus University, [b]Dept. of Environmental Science, Aarhus University, [c] Dept. Of Economics, Swedish University of Agricultural Sciences, [d]Baltic Study Centre, [e]Finnish Environment Institute (SYKE)

Supplementary Material S1

To discuss and validate findings with stakeholders and policy makers we conducted a series of four focus group and a pan-Baltic workshop in 2019.

Focus groups with invited policy makers and stakeholders were held by partners to the Tools2Sea project in Denmark (7 participants), Sweden (2 participants), Finland (4 participants) and Latvia (8 participants) in the period May-October 2019. Participants represented key stakeholder associations, including farmers associations, advisors, environmental NGO's, and additionally representatives from relevant ministries and agencies. Although, the four countries are not representative of the Baltic Sea catchment as such they reflect some of the opposites in terms of the intensity of farming systems, history of and approach to nutrient management. Discussions in the focus groups seeked to clarify national challenges in nutrient management and governance, including stakeholders perceptions of successful strategies to mitigate diffuse nutrient loss and shortcomings in the national approach. As these discussions were allowed to be context-sensitive, each session did not address the same themes. Following discussions, the main points from each focus group were summarized in a report prepared by the national teams. Subsequently, the four individual reports were merged in a joint synthesis that presented commonalities and divergence across the four countries.

After completing a draft of the article, the main findings were discussed at a pan-Baltic stakeholder workshop, which was held in Berlin November 2019. In support of the workshop, a short abstract with the main findings of the paper was prepared and circulated among participants. The workshop was open to all, but placed back-to-back with a meeting in the HELCOM Group on Sustainable Agricultural Practices (the AGRI group), which prepare and discuss HELCOM related policies regarding diffuse nutrient management. Participants to the workshop included policy makers with a focus on nutrient management, various technical experts and a range of stakeholders that engage policy development at a Baltic Sea level. In addition to the project partners, 25 participants attended the workshop.

The workshop was structured in two parts. In the first part, the initial findings of the BONUS Tools2Sea project where presented for the stakeholders in short 20 minutes presentations with opportunities for stakeholders to provide short feedback and ask clarifying questions. In the second part, workshop participants discussed opportunities to mitigate diffuse nutrient loss to the Baltic Sea in four heterogeneous focus groups. Discussions were facilitated by a moderator and were organized around three overall themes:

- Important and worthwhile addition to BSAP in terms of measures or specific policy instruments to reduce nutrient loading.
- What kind of support and from where, could different public authorities in BSR benefit from for fulfilling the targets and objectives of BSAP to reduce nutrient pollution?
- What are or could be appropriate mechanisms for fairness improving economic transfers to reduce nutrient loading and what are their pros and cons?

After the discussion, findings were presented in plenum and briefly discussed. After the workshop all four moderators prepared a summary of the key findings, which was synthesized in a joint internal report.
